# Rapid and robust association mapping of expression quantitative trait loci

**DOI:** 10.1186/1753-6561-1-s1-s144

**Published:** 2007-12-18

**Authors:** Alex C Lam, Michael Schouten, Yurii S Aulchenko, Chris S Haley, Dirk-Jan de Koning

**Affiliations:** 1 Department of Genetics and Genomics, Roslin Institute (Edinburgh), Edinburgh, Midlothian EH25 9PS, UK; 2 Department of Epidemiology and Biostatistics, Erasmus MC, 3000 CA Rotterdam, The Netherlands

## Abstract

We applied a simple and efficient two-step method to analyze a family-based association study of gene expression quantitative trait loci (eQTL) in a mixed model framework. This two-step method produces very similar results to the full mixed model method, with our method being significantly faster than the full model. Using the Genetic Analysis Workshop 15 (GAW15) Problem 1 data, we demonstrated the value of data filtering for reducing the number of tests and controlling the number of false positives. Specifically, we showed that removing non-expressed genes by filtering on expression variability effectively reduced the number of tests by nearly 50%. Furthermore, we demonstrated that filtering on genotype counts substantially reduced spurious detection. Finally, we restricted our analysis to the markers and transcripts that were closely located. We found five times more signals in close proximity (*cis*-) to transcripts than in our genome-wide analysis. Our results suggest that careful pre-filtering and partitioning of data are crucial for controlling false positives and allowing detection of genuine effects in genetic analysis of gene expression.

## Background

Association mapping is commonly used in detecting quantitative trait loci (QTL). For analyzing data collected from pedigrees, transmission disequilibrium test-based methods [1] are often an appropriate choice because they utilize only the within-family variation and, thus, are robust in the presence of population stratification. Alternatively, in carefully chosen study populations in which population stratification can be safely ruled out, measured genotype approaches [2] that exploit both the variation between- and within-family are expected to be the most powerful approaches for family-based association mapping. However, measured genotype approaches are time-consuming and therefore impractical for genome-wide association of multiple quantitative traits (such as global gene expression) due to the need to solve a large number of mixed model equations.

Another major challenge in this type of analysis is the massive inflation in the false positive (type I error) rate due to multiple-testing. Reducing the number of tests is one obvious way to control the number of false-positive results. In addition, many researchers have opted for the use of false-discovery rates (FDR) [3] to monitor the proportion of false positives amongst all positives. Nonetheless, balancing the control in type I and type II errors is a problematic issue in whole-genome analysis.

In this article, we present our analysis of the Genetic Analysis Workshop 15 (GAW15) gene expression data set (Problem 1) originating from Morley et al. [4]. We conducted family-based association mapping using data from all individuals to demonstrate the use of a two-step method [5] as a fast implementation of the mixed model approach. We applied two filtering methods to reduce multiple-testing and to discard a considerable number of spurious hits. In addition, we explored an alternative way to tackle multiple-testing and potentially improve detection by applying a separate analysis for *cis*-acting expression QTL (eQTL).

## Methods

### Pre-processing of data

All microarray files were pre-processed by "GCRMA" from the Bioconductor Project  version 1.8.0. From the 2882 SNPs provided, 2695 were selected because these were polymorphic among the individuals genotyped.

### Filtering on variability of the probe sets

Genes that are not expressed are not relevant to this study. Signal levels for non-expressed genes are typically above zero due to the background signals and other inherent systematic noises. Nonetheless, such genes can be detected on the basis that the background variation tends to be much less than real biological variation across samples. We adopted the interquartile range (IQR) as a measure of variability and used IQR of 0.1 as the threshold for this data set.

### Statistical method

The full mixed model for detecting marker association can be written as:

*y *= *Wa *+ *Xb *+ *Zu *+ *e*.

In Eq. (1), *y *is the expression trait values, *a*, *b*, *u*, and *e *are vectors of marker effect, other fixed effects (sex and generation), additive polygenic effect (random), and random residuals, respectively. *W*, *X*, and *Z *are incidence matrices related to marker, fixed, and polygenic effects, respectively.

The fast and robust method proposed by Aulchenko et al. is composed of two steps; the first step accounts for the familial dependence among family members and covariates of nuisance effects, and the second step tests the single SNP (single-nucleotide polymorphism) effect on the remaining variation by analysis of variance (ANOVA).

**Step 1: **For the expression values of each probe set we fitted the following mixed model without the marker effect:

*y *= *Xb *+ *Zu *+ *e*.

We fitted the models using ASReml  version 1.0. Narrow-sense heritability (*h*^2^) was estimated for each expression trait using the -*P *option in ASReml.

**Step 2: **Using the residuals from Step 1 as the new quantitative traits, the marker genotype effect of each SNP on each trait was tested by ANOVA. We used the lm() and anova() functions in R  version 2.3.1. FDR was calculated using the approach proposed by Storey and Tibshirani as implemented in the R package "*q*-value" [6].

### Detection of *cis*-acting eQTLs

eQTLs that associate with transcripts within 1 Mb of themselves are considered as *cis*-acting. Besides conducting the analysis at genome-wide level, we isolated a subset of 8462 probable *cis*-acting candidates (expression trait-SNP pairs), which comprised 2066 SNPs and 2797 expression traits, for mapping *cis*-acting eQTL separately. This was a much smaller search space and FDR was applied separately to obtain a new, group-wise significance threshold.

### Comparison of two-step method to the full mixed model method

We sampled 10,000 expression trait-SNP combinations for comparing the performance of the two-step approach and the full mixed model. Tests using the full mixed model described above were conducted using ASReml.

## Results and discussion

### Equivalence of the two-step method and the full mixed model method

**Figure 1:**
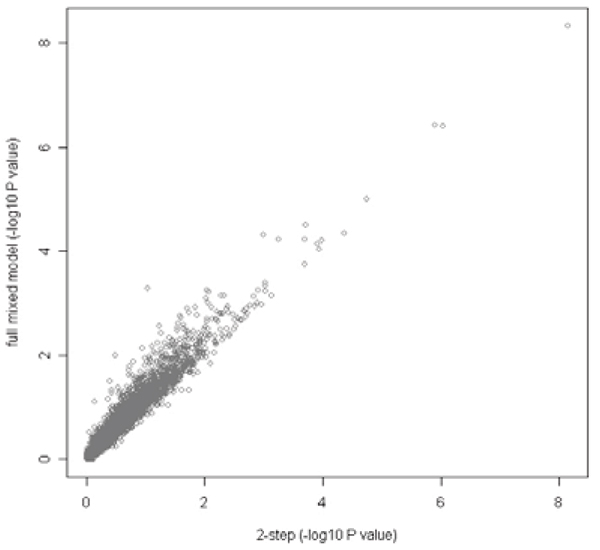
**Comparison of the two step and the full mixed model methods**. Transformed (-log_10_) *p*-values of 10,000 samples are plotted.

Our two-step method produced very similar *p*-values of the marker effect to the full mixed model (Fig. 1). For the GAW15 data set, we estimated a six-fold increase in speed with the two-step approach compared to the full mixed model approach using ASReml with our computing resources.

### Reduction of number of tests by filtering on variability

**Figure 2:**
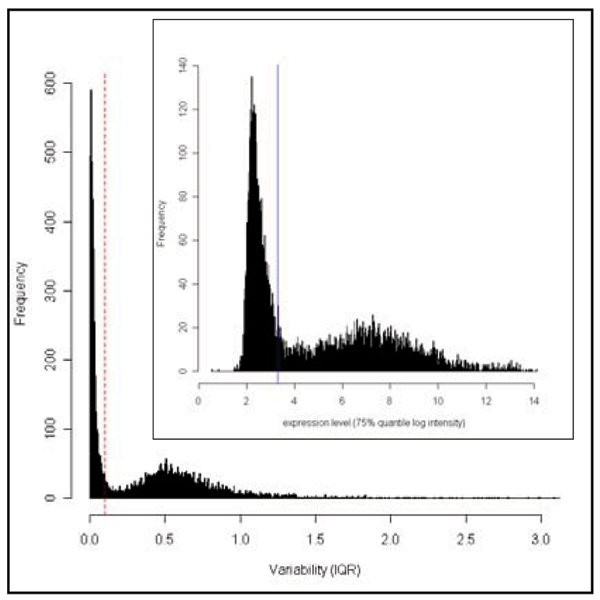
**Variability and expression level of expression traits**. Frequency distribution of the IQR of transcripts. The red dashed line indicates IQR of 0.1. The inserted histogram shows the expression level, as measured by the 75% quantile of each expression trait. All of the expression traits with IQR under 0.1 are found to be also lowly expressed, with the maximum expression level of 3.3 in log intensity (as indicated by the blue line).

Figure 2 (main) shows a large cluster of expression traits that has very low variability, and the inset shows a large cluster of expression traits with low log intensity (0–4). We used IQR of 0.1 as a cut off because expression traits below this threshold had low variability as well as low expression level. As a result, the number of probe sets was dramatically reduced from 8739 to 4627 (47%).

**Figure 3:**
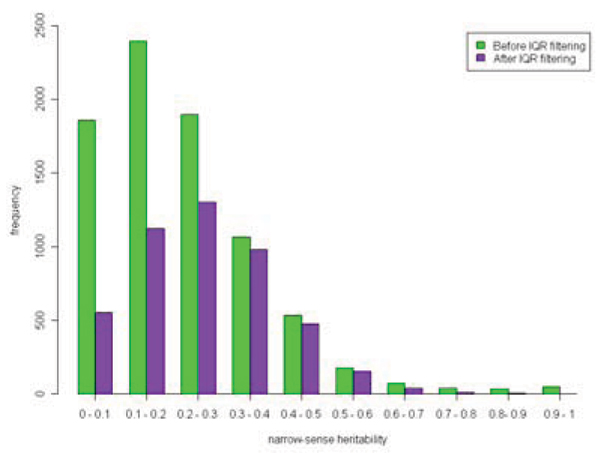
**Heritability of expression traits**. IQR filtering removed mostly the expression traits with low heritability.

The effect of removing non-expressed genes was roughly mirrored by the heritability distribution. By definition, heritability is a measure of the degree of genetic control of a trait and thus major eQTL detected for traits of low or zero heritability are unlikely to be real. It was reassuring that most expression traits filtered out were of low heritability (Fig. 3). By removing expression traits that have no biological relevance to the study, this filter substantially reduced multiple-testing and so potentially increased the power of our analysis to detect real eQTL.

### Numerous spurious associations in the initial analysis

**Figure 4:**
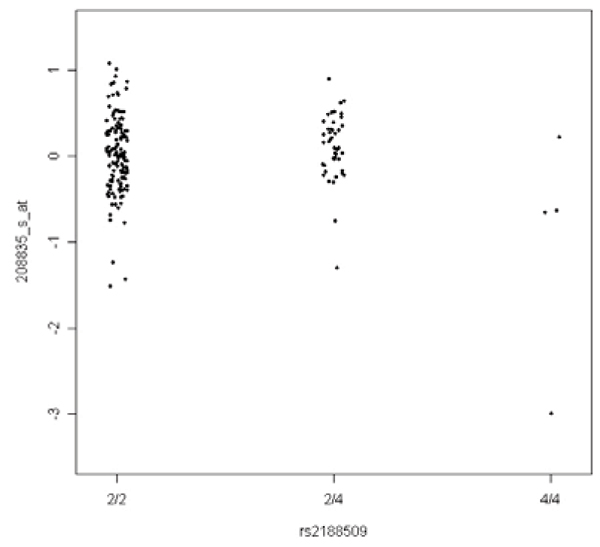
**Scatter plot of the expression trait residuals of probeset 208835_s_at after Step 1**. Spurious *p*-value of 2.6 × 10^-5 ^is caused by an outlier in genotype class 4/4.

Using the two-step approach described in the Methods, we detected 2282 associations at 20% FDR (*p *cut-off = 3.65 × 10^-5^). We observed that many significant hits were associated with the same SNPs. Although this phenomenon could be interpreted as some loci being the master regulator for a large number of transcripts, there is evidence that these vertical bands are likely to be artifacts. Table 1 shows the relationship between the number of significant associations and the sample size in the minor genotype class of a SNP. The SNPs with the most associations (with over 100 transcripts) were those with only one or two individuals in the minor genotype class. Conversely, we did not find SNPs with higher minor genotype count associated with multiple transcripts to the same extent. As ANOVA compared the phenotypic means of the genotype classes, outliers in the expression traits could have a big effect on the phenotypic mean, especially for SNPs which have genotype classes with a very small number of individuals. Figure 4 illustrates an example of such artifacts.

**Table 1:** Relationship between the minor genotype count and number of significant associations without the filtering of SNPs on genotype counts

Minor genotype count	No. SNPs	No. hits	Max. no. hits by a single SNP	Avg. no. hits per SNP
1	103	1054	200	10.23
2	55	333	147	6.05
3–6	166	508	48	3.06
7–10	56	107	12	1.91
11–15	52	85	9	1.63
16–20	42	56	4	1.33
21–30	45	65	5	1.44
>30	51	74	6	1.45

### Reduction in spurious associations by filtering on genotype counts

Subsequently, we employed a screening strategy on the SNP data by excluding any genotype classes with four or fewer individuals. We found 423 SNPs possessing at least one such genotype class. When the ANOVA tests were repeated, only 61 associations were detected at 20% FDR (*p *cut-off = 9.78 × 10^-7^). This finding suggests that the vast majority of associations previously detected were due to small sample size in SNP genotype classes, and therefore, unreliable. Note that the *p*-value threshold for the same FDR was much lower after having avoided the detection of many putative artifacts. FDR estimation is strongly influenced by the distribution of the *p*-values. If a large number of spurious effects are present due to violation of the underlying assumptions of the test statistic, excessive detection of false positives will not be prevented by the use of FDR.

Our strategy to screen the SNPs on genotype counts is superior to the commonly used filter based on minor allele frequency (typical thresholds used are 3, 5, or 10%). The latter approach is not sensitive to detect SNPs with a small genotype class because rare homozygous genotypes can be observed with minor alleles of moderate frequency under Hardy-Weinberg equilibrium, given the sample size of the current study.

It is also important to note that we only masked out genotype classes with small number of individuals rather than omitting all of the data for such SNPs. This has the advantage that the information from the remaining genotype classes could still be used for the tests. For example, having masked out the rare 4/4 (three individuals) genotype class from SNP rs1491846, its association with probeset 204133_at was detected at *p *= 1.13 × 10^-7^. Hence, our screening method is not only effective in excluding spurious effects, but also preserves genuine effects in the presence of rare genotype classes.

### Detection of *cis*-acting loci

Out of the 61 eQTLs detected, 3 eQTLs are within 1 Mb of their transcripts (*cis*-acting eQTL). Detecting so few *cis*-acting eQTLs is perhaps not a surprise because the SNP density in this data set is very low for whole-genome association mapping. Much of the genome would not be in strong linkage disequilibrium with the SNPs used in the genome scan. Effectively, only a small proportion of the genome has been screened. On the other hand, the tests for *cis*-acting eQTL are a tiny proportion of the total number of tests performed genome-wide. Therefore, they are heavily penalized by multiple-testing in the analysis above. Subsequently, we restricted the testing to only the SNPs and transcripts that were less than 1 Mb away from each other. This gave rise to 8462 *cis*-acting "candidates" (0.07% of all tests). At 20% FDR (*p *cut-off = 3.54 × 10^-4^), this analysis led to detection of an additional 12 *cis*-acting eQTLs (15 in total). Without laboratory-based validation, it is difficult to conclude whether partitioning the data in this way can increase power of detecting real *cis*-acting eQTL. Nonetheless, we would consider this strategy a practical way for improving the chance of detecting real *cis *effects. Because of the technical, statistical limitations and uncertainties in studying *trans*-regulation as described by Pastinen et al. [7], one may wish to dedicate more resources on studying *cis*-acting eQTL over *trans*-acting eQTL. This strategy increases the detection of *cis *signals and provides more "prioritized" candidate loci. In the present study, the number of candidates generated is still practically feasible to be followed up in laboratories.

### Findings compared with those of Morley et al. [4]

Our study did not identify the 13 eQTLs with the strongest linkage signals presented in Table 1 of Morley et al. [4]. However, given the differences in power of linkage and association studies and the low marker density for association, the results are not comparable. Alternative microarray pre-processing procedures and significance thresholds may also contribute to the differences.

## Conclusion

The two-step approach presented here is simple, fast, and efficient for family-based association studies in a mixed model framework. The speed advantage makes this implementation an attractive method for analyzing genome-wide association with large number of quantitative phenotypes. Filtering on variability of the probe sets dramatically reduces the number of expression traits and multiple-testing. Our method for masking rare genotype classes substantially decreases the number of spurious detection due to phenotypic outliers. Finally, limiting the search to SNPs and transcripts that are in close proximity appears to be a practical approach to avoid the excessive penalty imposed by multiple-testing on *cis*-acting eQTL and to increase the chance of detecting real signals for *cis*-regulation.

## Competing interests

The author(s) declare that they have no competing interests.
